# Iterative K-Closest Point Algorithms for Colored Point Cloud Registration

**DOI:** 10.3390/s20185331

**Published:** 2020-09-17

**Authors:** Ouk Choi, Min-Gyu Park, Youngbae Hwang

**Affiliations:** 1 Department of Electronics Engineering, Incheon National University, Yeonsu-gu, Incheon 22012, Korea; 2 Korea Electronics Technology Institute, Seongnam-si, Gyeonggi 13488, Korea; mpark@keti.re.kr; 3 Department of Electronics Engineering, Chungbuk National Univerisity, Cheongju, Chungbuk 28644, Korea

**Keywords:** registration, ICP, soft matching, point-to-plane, depth refinement

## Abstract

We present two algorithms for aligning two colored point clouds. The two algorithms are designed to minimize a probabilistic cost based on the color-supported soft matching of points in a point cloud to their *K*-closest points in the other point cloud. The first algorithm, like prior iterative closest point algorithms, refines the pose parameters to minimize the cost. Assuming that the point clouds are obtained from RGB-depth images, our second algorithm regards the measured depth values as variables and minimizes the cost to obtain refined depth values. Experiments with our synthetic dataset show that our pose refinement algorithm gives better results compared to the existing algorithms. Our depth refinement algorithm is shown to achieve more accurate alignments from the outputs of the pose refinement step. Our algorithms are applied to a real-world dataset, providing accurate and visually improved results.

## 1. Introduction

RGB-depth (RGB-D) cameras have recently been used in many application areas in robotics and 3D modeling due to their reduced costs and ease of use. A dense 3D map can be built by taking an RGB-D video of an environment and aligning the point clouds obtained from the RGB-D images [1,2]. An object’s 3D model can be obtained by using a multi-view system consisting of multiple calibrated RGB-D cameras [3,4]. The Iterative Closest Point (ICP) algorithm [5,6,7] plays an important role in the alignment [1,2] and compensation of the calibration error [3].

The ICP algorithm alternates between correspondence-search and pose-estimation steps to align two point clouds. Over the past thirty years, researchers have developed algorithms for overcoming the problems of the original algorithm [5]. To reduce the ambiguity in finding correspondences relying only on 3D location, the correspondence-search step has evolved to find the closest point in a higher-dimensional space, utilizing both 3D location and color [8,9,10]. Due to the limited resolution of the point clouds, a point may not be able to find its exactly corresponding point. A remedy to this problem is to allow a point to match all points in the other point cloud and assign matching probabilities to all the correspondences [11,12]. Another important line of research is to improve the cost function by replacing point-to-point distances with point-to-plane or plane-to-plane distances [6,13]. By alleviating the assumption of point-to-point correspondence, the algorithms become more accurate and robust.

The first contribution of this paper is a novel probabilistic cost function based on the color-supported soft matching of points to their *K*-closest points. The depth values measured by RGB-D cameras suffer from errors [14,15,16], which hinder obtaining accurate one-to-one correspondences. The probabilistic one-to-many correspondences help to improve the pose accuracy in the presence of the measurement errors. The probability is defined as the similarity in color and 3D position between points to reduce the matching ambiguity. Finally, our cost function is defined as the weighted sum of the squared point-to-point and point-to-plane distances, where the probability is used as the weight. The mixed form of the cost function is stable to minimize and produces robust and accurate results. To our best knowledge, a cost function in this form has not been reported yet.

Based on the cost function, we propose two different algorithms. The first algorithm, like prior ICP algorithms, finds the pose parameters that minimizes the cost. The second contribution of this paper is our second algorithm regarding the measured depth values as variables and the pose parameters as constants. In our second algorithm, the cost is minimized by refining the depth values so that the two point clouds get aligned more closely.

In this paper, we are interested in reconstructing the 3D structure of a person using multiple RGB-D cameras surrounding the person. The RGB-D cameras are assumed to be calibrated, but their pose parameters are assumed to suffer from errors due to inaccurate calibration [3] or unfixed camera locations. To our knowledge, no publicly available RGB-D dataset in this multi-view setting provides ground truth pose parameters and depth values. Building such a dataset will require several high-end laser scanners accurately calibrated with the RGB-D cameras.

Another contribution of this paper is a synthetic multi-view RGB-D dataset used for in-depth evaluation of our algorithms. We build the dataset by rendering computer graphics human models [17] and injecting realistic depth noise. Experiments show that our pose refinement algorithm gives better results compared to the existing methods. Our depth refinement algorithm is shown to reduce the registration error further, achieving more accurate alignments. The two algorithms are also applied to a real-world dataset, delivering accurate and visually improved results.

The remainder of this paper is structured as follows. The next section provides a summary of existing methods. Section 3 presents our proposed algorithms. The algorithms for applying the proposed algorithms to multi-view point-cloud registration are described in Section 4. Our new synthetic dataset is described in Section 5, followed by a subsequent section providing experimental results. Finally, Section 7 concludes the paper.

## 2. Related Work

In this paper, we assume that the input point clouds are nearly aligned. If the initial alignment is arbitrary, practical registration pipelines use a global registration algorithm [18,19,20,21] to compute the pose. The pose is then further refined by a local registration algorithm like the ICP algorithm [5]. For the global registration, Aiger et al. [18] exploit geometric invariants of affine transformations to produce the initial pose hypotheses, which are integrated into the RANSAC algorithm [22]. Mellado et al. [19] propose a smart indexing data organization for accelerating this method. Instead of random sampling [22], a branch-and-bound scheme can be used to systematically explore the pose space in search of the optimal solution [20]. Zhou et al. [21] use histogram features [23] to establish candidate matches. For fast computation, their algorithm operates only on the candidate matches and minimizes a robust cost function using the graduated non-convexity algorithm without correspondence updates or closest-point queries.

To our knowledge, the ICP algorithm was originally proposed by three different groups [5,6,7] almost simultaneously. The ICP algorithm proposed by Besl and McKay [5] is recognized as the standard original algorithm, consisting of correspondence-search and pose-estimation steps. The algorithm of Chen and Medioni [6] minimizes the sum of squared point-to-plane distances, and this cost function is still employed by state-of-the-art methods [24].

In the correspondence search step [5], a point in a point cloud finds the closest point in the other point cloud. Only the matching pairs, of which the distance is shorter than a threshold [5], are used in the pose estimation step. A low threshold level tends to fail to collect sufficient matching pairs while a high threshold level tends to fail to reject outliers. The algorithm of Zhang [7] adjusts the threshold level according to data statistics. To avoid the heuristic decision of the threshold level, Fitzgibbon [25] uses a robust loss function, and Bouaziz et al. [26] use sparsity inducing norms instead of the squared distances.

Since the original correspondence-search step relies only on the distance between points, the resulting correspondences are prone to error if the initial alignment is not close. As a remedy to this problem, researchers have devised methods that incorporate the color information in the correspondence search step [8,9,10]. The key idea is to join the 3D coordinates and the color of a point into a higher dimensional vector to perform a 4D [9] or 6D [8,10] search. Our pose refinement algorithm uses the correspondence search method of Johnson and Kang [8], extending the nearest neighbor search to the *K*-nearest neighbor search.

The two point clouds to be registered may be of different resolutions or low resolutions. In these cases, a point may not be able to find its exactly corresponding point. Furthermore, the correspodences in the early steps are innately inaccurate. Probabilistic approaches [11,12,27] allow a point to match all points in the other point cloud, assigning matching probabilities to all the correspondences. The approaches in [11,12] use loose probabilities that reflect the matching uncertainty in the early steps. In the final steps, the probabilities are tightened so that the closest point will be assigned a higher probability. This annealing scheme forms a smoothed cost function that is easier to minimize in the early steps. The pose is gradually refined to reach the neighborhood realm of the true pose as the cost function becomes more accurate with the tightened probability. These fully probabilistic approaches [11,12,27] are computationally demanding if the number of points is large. Although the approach in [27] uses the distance threshold to reduce the number of correspondences, the number may not decrease much if the distance threshold is high. As a trade-off, we assign the probabilities only to the *K*-closest points, which can be efficiently obtained using a *K*D tree [28]. In addition, we rely on a coarse-to-fine scheme [24], which is fast and effective, instead of the annealing scheme.

An effective way to reduce the registration error is to use an improved cost function that does not imply point-to-point correspondence. The Euclidean distance in the original cost function [5] can be modified into a Mahalanobis distance using a 3×3 matrix. Segal et al. [13] show that the Mahalanobis distance can represent both point-to-plane and plane-to-plane distances by only changing the matrix. The Mahalanobis distance can also be used to reflect the anisotropic, inhomogeneous localization error of the measured points [29]. Park et al. [24] use a cost function based on both color and depth differences between two point clouds instead of performing a higher dimensional search.

Deformable ICP algorithms have been developed to change the surface as well as the pose to achieve closer alignments [30,31,32]. The algorithm of Sinha et al. [30] employs a statistical shape model [33], which is obtained from a number of deformed shapes of a class of objects. Although we are interested in reconstruction of humans, we do not want to restrict our algorithm to a specific object class. Thus our algorithms do not rely on the statistical shape model. The algorithms in [31,32] compute a displacement field based on regularization terms for smoothing the field. Our depth refinement algorithm also uses a regularization term. The regularization is, however, not just for smoothing the displacement field but also for moving the points without a corresponding point.

Our depth refinement algorithm is similar to a depth noise reduction method [15] in that a depth-update equation is derived. The main difference is that our refinement is across point clouds, while the noise reduction in [15] is performed within a single depth image. In addition, the goal of our update equation is rather to align two point clouds more closely than to reduce the noise.

## 3. Iterative K-Closest Point Algorithms

In this section, we present our iterative *K*-closest point algorithms for registering a source point cloud to a reference point cloud. The two point clouds are assumed to be obtained from RGB-D images, which can be acquired with RGB-D cameras or stereo cameras.

### 3.1. Iterative K-Closest Point Algorithm for Pose Refinement

We present our algorithm for refining the rigid transformation from a source point cloud $S_{s}={\left\{X_{i}^{\left(s\right)}\right\}}_{i=1}^{N_{s}}$ to a reference point cloud $S_{r}={\left\{X_{i}^{\left(r\right)}\right\}}_{i=1}^{N_{r}}$. The transformation is parameterized by a 3×3 rotation matrix R and a 3D translation vector T such that

(1) $$X_{j}^{\left(r\right)}=RX_{i}^{\left(s\right)}+T$$

for a corresponding pair $X_{i}^{\left(s\right)}$ and $X_{j}^{\left(r\right)}$. We follow the general ICP framework, which alternates between correspondence-search and pose-estimation steps. One of the differences in our algorithm from the original ICP algorithm [5] is that the correspondence is probabilistic. Unlike the fully probabilistic approaches [11,12], we assign matching probabilities only to the *K*-closest points. Setting *K* to a large number will increase the computational complexity while setting it to a small number will not produce a smoothed cost function that is easy to minimize [11,12]. In this paper, we set *K* to 5 unless otherwise mentioned.

In the correspondence-search step, we perform a 6D search based on both 3D location and color [8]. Each point $X_{i}^{\left(s\right)}$ in $S_{s}$ is transformed to ${\hat{X}}_{i}^{\left(s\right)}=RX_{i}^{\left(s\right)}+T$ by using the current pose parameters R and T. Since we assume that the point clouds are obtained from RGB-D images, each point $X_{i}^{\left(s\right)}$ is associated with its color vector $C_{i}^{\left(s\right)}$. ${\hat{X}}_{i}^{\left(s\right)}$ and $C_{i}^{\left(s\right)}$ are joined together to produce a 6D vector $\left({\hat{X}}_{i}^{\left(s\right)},\beta \circ C_{i}^{\left(s\right)}\right)$, where $C_{i}^{\left(s\right)}$ is represented in the YIQ color space and then multiplied by a weight vector β for balancing between the two different quantities [8]. The operation “∘” represents the Hadamard product. Such 6D vectors ${\left\{\left(X_{i}^{\left(r\right)},\beta \circ C_{i}^{\left(r\right)}\right)\right\}}_{i=1}^{N_{r}}$ are obtained from the reference points to build a *K*D tree [28] for accelerating the 6D search. The *K*-nearest neighbors to $\left({\hat{X}}_{i}^{\left(s\right)},\beta \circ C_{i}^{\left(s\right)}\right)$ are then searched for, using the *K*D tree.

Denoting the set of the *K*-nearest neighbor indices as $N_{i}$, the *K*-closest points are $X_{j}^{\left(r\right)}$ for $j\in N_{i}$. The residual vector $d_{i,j}$ between ${\hat{X}}_{i}^{\left(s\right)}$ and $X_{j}^{\left(r\right)}$ is defined as

(2) $$d_{i,j}=X_{j}^{\left(r\right)}-{\hat{X}}_{i}^{\left(s\right)}=X_{j}^{\left(r\right)}-RX_{i}^{\left(s\right)}-T.$$

Likewise, we define a 6D difference vector $c_{i,j}$ as

(3) $$c_{i,j}=\left(X_{j}^{\left(r\right)},\beta \circ C_{j}^{\left(r\right)}\right)-\left({\hat{X}}_{i}^{\left(s\right)},\beta \circ C_{i}^{\left(s\right)}\right).$$

We assume that $N_{i}$ has been sorted in ascending order of $∥c_{i,j}∥$. The first element in $N_{i}$, which is the nearest neighbor index, is denoted as $a_{i}$ for later use.

In the pose-estimation step, we find R and T that minimize the following cost:

(4) $$E=\frac{1}{2}\sum_{i=1}^{N_{s}}\sum_{j\in N_{i}}p_{i,j}d_{i,j}^{T}M_{i,j}d_{i,j},$$

where $p_{i,j}$ is the matching probability between $X_{i}^{\left(s\right)}$ and $X_{j}^{\left(r\right)}$ such that either $\sum_{j\in N_{i}}p_{i,j}=1$ or $p_{i,j}=0$ for all $j\in N_{i}$. $M_{i,j}$ is a 3×3 matrix for defining the cost function as the sum of squared Mahalanobis distances in an arbitrary form. The factor $\frac{1}{2}$ is for eliminating the factor 2 produced by differentiating the quadratic function.

Based on the assumption that the corresponding point is close to ${\hat{X}}_{i}^{\left(s\right)}$ in terms of both 3D location and color, we define $p_{i,j}$ as

(5) $$p_{i,j}=\left\{\begin{array}{cc} \gamma_{i}\exp\left(-\frac{∥c_{i,j}{∥}^{2}}{2\sigma_{c}^{2}}\right), & \mathrm{if}∥c_{i,j}∥<\tau_{c},\\ 0, & \mathrm{otherwise}, \end{array}\right.$$

where $\gamma_{i}$ is the normalizing coefficient for ensuring the sum of $p_{i,j}$ to be 1 unless all values of $p_{i,j}$ are 0. $\tau_{c}$ is the distance threshold typically employed in most ICP algorithms. In this paper, we use a user-defined value, which will be specified later in this section. If the median of $\{∥c_{i,a_{i}}{∥\}}_{i=1}^{N_{s}}$ is greater than $\tau_{c}$ for the initial pose parameters $R^{0}$ and $T^{0}$ then $\tau_{c}$ is replaced with the median. This is intended to provide a sufficient number of correspondences if the initial registration error is large. $\sigma_{c}$ is set to $\tau_{c}$.

Throughout this paper, we assume that the initial pose parameters $R^{0}$ and $T^{0}$ are given. In practice, we can employ global registration algorithms [18,19,20,21] to estimate them. For a multi-view system, they can be estimated by calibrating the camera network [34].

The state-of-the-art cost functions are defined as the sum of squared point-to-plane [6,24] or plane-to-plane distances [10,13]. For the plane-to-plane distances, $M_{i,j}$ depends on both source and reference points and needs to be recomputed after every correspondence-search step. In contrast, for the point-to-plane distances, $M_{i,j}$ depends only on reference points and needs to be computed only once. In this case, $M_{i,j}$ is defined as

(6) $$M_{i,j}=n_{j}n_{j}^{T},$$

where $n_{j}$ is the surface normal vector of $X_{j}^{\left(r\right)}$.

The rank of $n_{j}n_{j}^{T}$ is 1 and thus $n_{j}n_{j}^{T}$ is noninvertible. To increase the numerical stability of our optimization algorithm, we add ϵI to $n_{j}n_{j}^{T}$, where ϵ is a small positive number (0.001 in this paper). Our $M_{i,j}$ is defined as

(7) $$M_{i,j}=M_{j}^{\left(r\right)}=\epsilon I+n_{j}n_{j}^{T}.$$

Defining $M_{i,j}$ in this form is also equivalent to defining the cost function as the weighted sum of squared point-to-point and point-to-plane distances.

To minimize *E* in Equation (4), we apply the Gauss–Newton algorithm, which is widely employed in ICP variants [24,25,35]. During the minimization, $p_{i,j}$ is regarded as a fixed variable although it depends on the pose parameters. We use the approximation form for an incremental rotation [35] as defined by

(8) $$\Delta R=\left[\begin{array}{ccc} 1 & -\Delta \theta_{Z} & \Delta \theta_{Y}\\ \Delta \theta_{Z} & 1 & -\Delta \theta_{X}\\ -\Delta \theta_{Y} & \Delta \theta_{X} & 1 \end{array}\right],$$

where $\Delta \theta_{X}$, $\Delta \theta_{Y}$, and $\Delta \theta_{Z}$ are the rotation angles about *X*, *Y*, and *Z* axes, respectively. Denoting an incremental translation by $\Delta T=(\Delta T_{X},\Delta T_{Y},\Delta T_{Z})$, the incremental transformation parameters can be represented by a vector $\delta ={\left(\Delta \theta_{X},\Delta \theta_{Y},\Delta \theta_{Z},\Delta T_{X},\Delta T_{Y},\Delta T_{Z}\right)}^{T}$.

Let us define $d_{i,j}\left(0\right)$ as $d_{i,j}$ computed with the current rotation and translation parameters. $d_{i,j}\left(\delta \right)$ is then approximated by

(9) $$d_{i,j}\left(\delta \right)\approx d_{i,j}\left(0\right)+J_{i}\delta ,$$

where $J_{i}$ is the 3×6 Jacobian matrix with the partial derivatives of $d_{i,j}$ with respect to the components of δ:

(10) $$J_{i}=\left[\begin{array}{cccccc} 0 & -{\hat{Z}}_{i}^{\left(s\right)} & {\hat{Y}}_{i}^{\left(s\right)} & -1 & 0 & 0\\ {\hat{Z}}_{i}^{\left(s\right)} & 0 & -{\hat{X}}_{i}^{\left(s\right)} & 0 & -1 & 0\\ -{\hat{Y}}_{i}^{\left(s\right)} & {\hat{X}}_{i}^{\left(s\right)} & 0 & 0 & 0 & -1 \end{array}\right],$$

The gradient of *E* is then computed as

(11) $$\nabla E=\sum_{i=1}^{N_{s}}\sum_{j\in N_{i}}p_{i,j}\left(J_{i}^{T}M_{i,j}J_{i}\delta +J_{i}^{T}M_{i,j}d_{i,j}\left(0\right)\right).$$

Solving for δ satisfying ∇E=0 gives

(12) $$\delta =-{\left(\sum_{i=1}^{N_{s}}J_{i}^{T}\left(\sum_{j\in N_{i}}p_{i,j}M_{i,j}\right)J_{i}\right)}^{-1}\left(\sum_{i=1}^{N_{s}}J_{i}^{T}\left(\sum_{j\in N_{i}}p_{i,j}M_{i,j}d_{i,j}\left(0\right)\right)\right)$$

The new rotation matrix and translation vector is computed as

(13) $$\begin{array}{cc} R & \leftarrow \Delta RR, \end{array}$$

(14) $$\begin{array}{cc} T & \leftarrow \Delta RT+\Delta T. \end{array}$$

${\hat{X}}_{i}^{\left(s\right)}$ is updated using the new pose parameters and the Gauss–Newton step is iteratively applied until convergence. The algorithm is configured to terminate when the magnitudes of the incremental rotation and translation are below threshold levels or when a maximum iteration count is reached. The thresholds are set to 0.001° rotation and 0.001 mm translation with a maximum iteration count of 80.

Algorithm 1 summarizes the presented algorithm for pose refinement.
**Algorithm 1:** Iterative *K*-closest point algorithm for pose refinement.
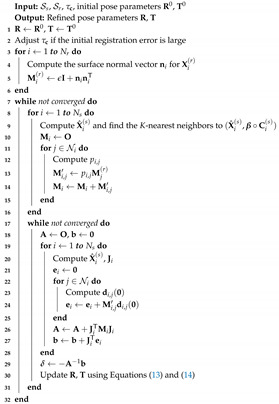


Since our pose refinement algorithm is a local registration algorithm, a good initial pose is necessary to converge to an accurate pose. To alleviate getting trapped in local minima, probabilistic approaches rely on annealing schemes [6,12]. In this paper, we rely on a coarse-to-fine scheme [24]. Three pairs of point clouds of different resolutions are built by downsampling input source and reference point clouds using voxel grids with voxel sizes of 4, 2, and 1 cm. The output from a coarse level is used as the input initial pose parameters to the adjacent finer level. Our assumption of small ΔR and ΔT may not hold in coarse levels because we cannot guarantee the quality of the initial alignment. Thus, we set $M_{i,j}$ to I in the two coarse levels. The resulting cost function can be minimized by finding the direct solution to a weighted least squares problem [36]. For each level, $\tau_{c}$ is set to $\sqrt{2}$ times the voxel size.

The downsampling in the coarse-to-fine scheme is similar to the decimation in the annealing scheme of the multiscale EM-ICP algorithm [12]. The scale parameter in [12] has a similar meaning to the voxel size. With a small number of data points at a coarse level, the shape of the cost function becomes simple and easy to minimize. Although the optimal solution of a coarse level tends to be shifted from the optimal solution to the original cost function [12], it can be a good initial solution for the next level. This coarse-to-fine scheme cannot deal with an arbitrary initial pose, but it was shown to be efficient and effective in practice [24].

### 3.2. Iterative K-Closest Point Algorithm for Depth Refinement

An interesting discovery of this paper is that the alignment between two point clouds can also be achieved by refining the measured depth values. Regarding the measured depth value $Z_{i}^{\left(s\right)}$ of a point $X_{i}^{\left(s\right)}$ as a variable, we can solve for $Z_{i}^{\left(s\right)}$ minimizing Equation (4). Regarding R and T as constants, *E* can be considered as the sum of independent cost functions $E_{i}$:

(15) $$E=\sum_{i=1}^{N_{s}}E_{i},$$

where

(16) $$E_{i}=\frac{1}{2}\sum_{j\in N_{i}}p_{i,j}d_{i,j}^{T}M_{i,j}d_{i,j}.$$

Denoting the normalized image coordinate vector [22] corresponding to $X_{i}^{\left(s\right)}$ as $x_{i}$, $X_{i}^{\left(s\right)}$ equals $Z_{i}^{\left(s\right)}x_{i}$. The gradient of $d_{i,j}$ with respect to $Z_{i}^{\left(s\right)}$ is computed as

(17) $$\nabla d_{i,j}=-Rx_{i}.$$

By applying the chain rule, the derivative of $E_{i}$ with respect to $Z_{i}^{\left(s\right)}$ is computed as

(18) $$\frac{dE_{i}}{dZ_{i}^{\left(s\right)}}=\sum_{j\in N_{i}}p_{i,j}\nabla^{T}d_{i,j}M_{i,j}d_{i,j}=-x_{i}^{T}R^{T}\left(\sum_{j\in N_{i}}p_{i,j}M_{i,j}\left(X_{j}^{\left(r\right)}-T-Z_{i}^{\left(s\right)}Rx_{i}\right)\right).$$

Solving for $Z_{i}^{\left(s\right)}=Z_{i,opt}^{\left(s\right)}$ satisfying $\frac{dE_{i}}{dZ_{i}^{\left(s\right)}}=0$ yields to

(19) $$Z_{i,opt}^{\left(s\right)}=\frac{x_{i}^{T}R^{T}\sum_{j\in N_{i}}p_{i,j}M_{i,j}\left(X_{j}^{\left(r\right)}-T\right)}{x_{i}^{T}R^{T}\left(\sum_{j\in N_{i}}p_{i,j}M_{i,j}\right)Rx_{i}}.$$

Algorithm 2 summarizes the presented algorithm for depth refinement. In the depth refinement step, we assume that the pose refinement algorithm has aligned the point clouds, so we perform the conventional 3D search in Algorithm 2. We use a spatial distance threshold $\tau_{d}=4$ cm to prevent a spatially distant point from becoming a neighbor to ${\hat{X}}_{i}^{\left(s\right)}$. However, we use the same weighting function in Equation (5) to allow similarly colored point pairs to have high $p_{i,j}$. In Equation (5), the condition $∥c_{i,j}∥<\tau_{c}$ is replaced with $∥d_{i,j}∥<\tau_{d}$ based on the 3D search.
**Algorithm 2:** Iterative *K*-closest point algorithm for depth refinement.
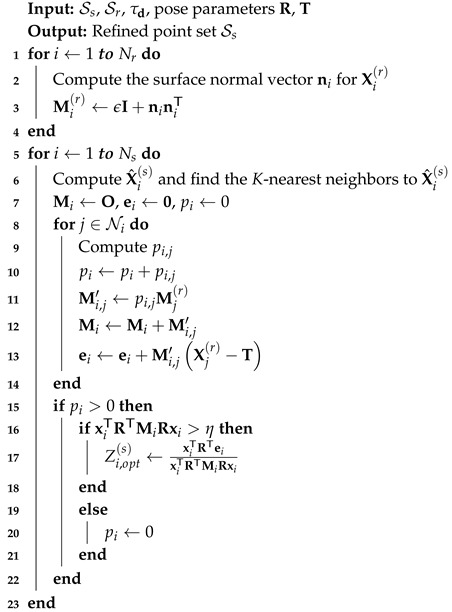


In Algorithm 2, η=0.1 is a positive constant for ensuring the numerical stability of the division. For some points, $|Z_{i,opt}^{\left(s\right)}-Z_{i}^{\left(s\right)}|$ can be greater than $\tau_{d}$. In this case, $Z_{i,opt}^{\left(s\right)}$ is set to either $Z_{i}^{\left(s\right)}+\tau_{d}$ or $Z_{i}^{\left(s\right)}-\tau_{d}$ to restrict the change to be local.

Not all source points may find their closest points within the threshold level. In this case, source points without valid closest points can not be changed by Algorithm 2. To attract the unchanged points to the refined points, we minimize the following cost *F* instead of directly replacing $Z_{i}^{\left(s\right)}$ with $Z_{i,opt}^{\left(s\right)}$.

(20) $$F\left({\left\{Z_{i}^{\left(s\right)}\right\}}_{i=1}^{N_{s}}\right)=\sum_{i=1}^{N_{s}}p_{i}{\left(Z_{i}^{\left(s\right)}-Z_{i,opt}^{\left(s\right)}\right)}^{2}+\frac{\lambda }{2}\sum_{i=1}^{N_{s}}\sum_{m\in R_{i}}{\left(Z_{i}^{\left(s\right)}-Z_{m}^{\left(s\right)}-\Delta Z_{i,m}\right)}^{2},$$

where $p_{i}$, which is either 1 or 0, is an indicator of valid $Z_{i,opt}^{\left(s\right)}$ for $Z_{i}^{\left(s\right)}$. $\Delta Z_{i,m}$ is the initial difference $Z_{i}^{\left(s\right)}-Z_{m}^{\left(s\right)}$ before minimizing *F*. $R_{i}$ is the eight neighbors’ indices around the pixel location of $X_{i}^{\left(s\right)}$ in the image.

Applying the Jacobi method to minimization of *F* yields to the following update equation:

(21) $$Z_{i}^{\left(s\right)}\leftarrow \frac{p_{i}Z_{i,opt}^{\left(s\right)}+\lambda \sum_{m\in R_{i}}\left(Z_{m}^{\left(s\right)}+\Delta Z_{i,m}\right)}{p_{i}+\lambda \left|R_{i}\right|},$$

where λ is a weight for balancing between the two different terms, which is set to 0.2 throughout this paper. $|R_{i}|$ is the number of elements in $R_{i}$. For $Z_{i}^{\left(s\right)}$ with $p_{i}=0$, a neighboring updated $Z_{m}^{\left(s\right)}$ pulls $Z_{i}^{\left(s\right)}$ toward $Z_{m}^{\left(s\right)}$ by an amount of $\Delta Z_{i,m}$ preserving the initial relative difference. The update equation is applied to all source points six times. This small maximum iteration count is appropriate if the source fragment is a proper subset of the reference fragment. For this reason, we apply the presented algorithm only to the merged point cloud described in the next section.

## 4. Multi-View Point-Cloud Registration

Given point clouds from multiple viewpoints, we iteratively merge neighboring point clouds and eventually merge all the multi-view point-clouds together. Although an advanced algorithm based on pose-graph optimization exists [37], we present a simpler method that is adequate for evaluating the robustness of the pairwise registration algorithm. Because the error in a single pair propagates to the error in the subsequent pairs, the robustness of the pairwise registration is important in this application.

Given a reference point cloud $S_{r}=S_{0}$ and its neighboring point cloud $S_{s}$, we can apply the method in Section 3.1 to register $S_{s}$ to $S_{r}$. To obtain the merged point cloud S, $S_{s}$ is transformed to ${\hat{S}}_{s}$ using the estimated pose parameters. S is then computed as the union of $S_{r}$ and ${\hat{S}}_{s}$. Regarding S as a new reference point cloud, a new neighboring point cloud is merged to S. This pairwise merging procedure is repeated until the farthest point cloud $S_{f}$ is merged.

Although the sequential merging procedure cannot prevent error accumulation, we make the following effort that can reduce the error. After one round of merging, we begin the procedure in reverse order with S being initially set to the union of $S_{0}$ and ${\hat{S}}_{f}$, which is the transformed $S_{f}$ to the coordinate frame of $S_{0}$. Because our pairwise registration algorithm is not symmetric, the solution can be different if the source and reference are reversed. By reversing the order, our algorithm gets a chance to refine the current pose with the reversed input pair. If the current pose is not accurate, the reversed input can help to escape from the local minimum. If a single forward path from $S_{0}$ to $S_{f}$ is accurate, then the pose of $S_{f}$ can be accurate. In this case, the error in the other reverse paths can be decreased by registering the erroneous point clouds to $S_{f}$ in its accurate position and orientation. However, if there exists only a single path to $S_{f}$ then it is hard to expect such error-correction.

Algorithm 3 summarizes the presented multi-view point cloud merging algorithm. Without loss of generality, the 0-th view is assumed to be the reference view. $ITER_{max}$ is set to five throughout this paper.
**Algorithm 3:** Multi-view point cloud merging algorithm.
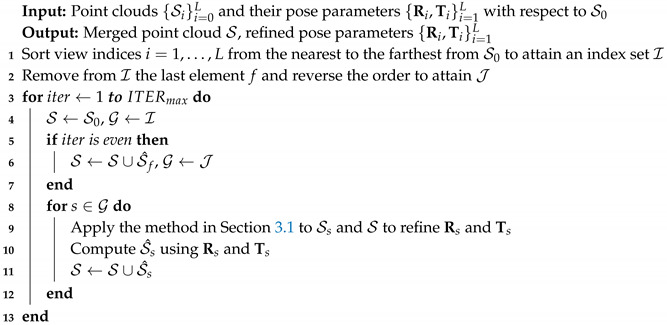


After merging the point clouds, we can apply the method in Section 3.2 to reduce the registration error further. At this stage, $S_{s}$ is registered to $S-{\hat{S}}_{s}$ by refining the depth values of $S_{s}$. The depth-refining process begins with the farthest point cloud $S_{f}$ and ends with the reference point cloud $S_{0}$ in the first round. The order is based on the assumption that the farthest point cloud will be the most erroneous. In the second round, the processing order is reversed.

Algorithm 4 summarizes the presented algorithm. Here, ${ITER}_{max}$ is set to two throughout this paper.
**Algorithm 4:** Multi-view depth refinement algorithm.
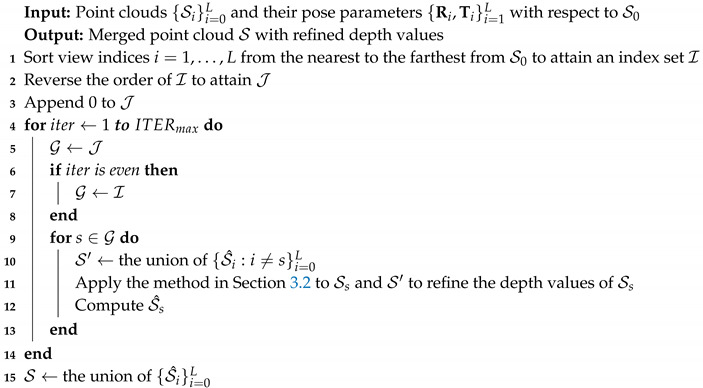


## 5. Synthetic Multi-View RGB-D Dataset

We build a synthetic multi-view RGB-D dataset by rendering graphics models of the pose-varying human model dataset [17]. We use a male (models 0–199) and a female (models 6800–6999) appearance and regularly sample 20 mesh models of different poses from each appearance. The height of the mesh models is set to 1.7 m.

**Figure 1:**
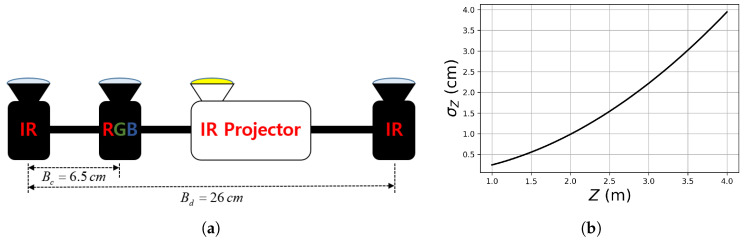
Our virtual active stereo camera and its depth measurement noise model. (**a**) Two IR cameras and an IR projector comprise an active stereo vision system. The left IR camera is the reference camera. The color camera is used to acquire RGB data. (**b**) The standard deviation $\sigma_{Z}$ of depth measurement error according to depth *Z*. Please refer to the text for more detail.

We assume that the RGB-D images are acquired by an active stereo camera depicted in Figure 1a. Two infrared (IR) cameras and an IR projector comprise an active stereo vision system for depth acquisition. The depth acquisition process is simulated by rendering the depth image of a mesh model and then adding realistic random errors to the depth values. We assume that the error in the disparities attained by stereo matching follows a zero-mean Gaussian distribution with a standard deviation of $\sigma_{d}=0.5$ pixel. The depth error then approximately follows a zero-mean Gaussian distribution, of which the standard deviation $\sigma_{Z}$ depends on the depth value *Z*:

(22) $$\sigma_{Z}=\frac{Z^{2}}{f_{d}B_{d}}\sigma_{d},$$

where $f_{d}$ and $B_{d}$ are the focal length and the baseline length of the stereo camera, which are set to 780 pixels and 26 cm, reflecting the real parameters used in Section 6. The width and height of the depth image are 1468 and 1228 pixels and the image center coordinate vector is (734, 614). Figure 1b shows $\sigma_{Z}$ according to *Z* from 1 m to 4 m.

The noisy depth image is forward-warped to the color-camera coordinate frame by using the intrinsic and extrinsic camera parameters to form an RGB-D image. The focal length of the color camera is 900 pixels and the distance from the reference IR camera is 6.5 cm. The color image has the same width, height, and image center as the depth image. The ground truth depth image is obtained by rendering the mesh model directly onto the color image coordinate frame.

**Figure 2:**
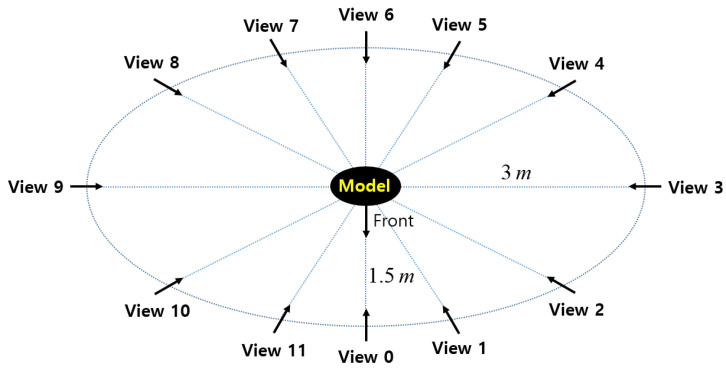
Multi-view Red Green Blue—Depth (RGB-D) camera setup for acquiring our synthetic dataset. The cameras are on an elliptical arc at $30^{\circ }$ intervals. The semi-major and semi-minor axes are 3 and 1.5 m, respectively.

We use twelve (i.e., L=11) virtual stereo cameras surrounding a mesh model to acquire RGB-D images from different viewpoints. The distance from the mesh model to the cameras varies from 1.5 to 3 m as depicted in Figure 2. The height of the cameras is 1.7 m and they look downward with a pitch angle of $20^{\circ }$. Since viewpoints 3 and 9 are far from the model, their acquired depth images are highly noisy, and the distance to their neighboring viewpoints is long. Therefore, pairwise registration involving the two views will be more challenging.

**Figure 3:**
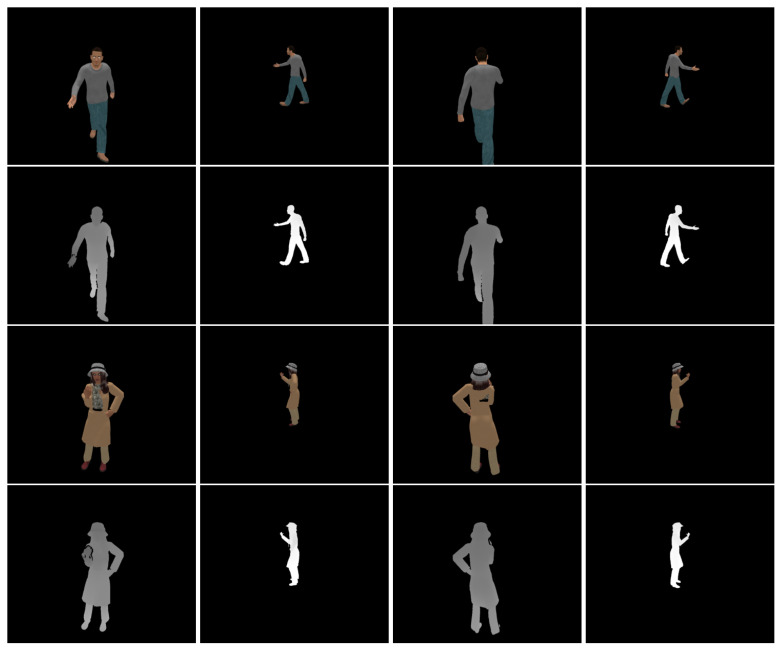
Sample RGB-D images in our synthetic dataset. **First row**: Color images of the male model. **Second row**: Depth images of the male model. **Third row**: Color images of the female model. **Fourth row**: Depth images of the female model. **First column**: View 0. **Second column**: View 3. **Third column**: View 6. **Fourth column**: View 9. The intensity of the depth images is linear with depth values.

Figure 3 shows sample RGB-D images acquired by the virtual stereo cameras. The depth images suffer from missing data due to self-occlusions, as observed in real depth images. Furthermore, the point resolution is uneven across views due to the different distances to the models.

The ground truth pose parameters ${\left\{R_{i,gt},T_{i,gt}\right\}}_{i=1}^{L}$ of the stereo cameras are perturbed to produce the initial inaccurate pose parameters, which are used as input to our method. To perturb the rotation matrix $R_{i,gt}$ we premultiply $R_{i,gt}$ by a rotation matrix ΔQ with a random axis of rotation.

(23) $$R_{i}=\Delta QR_{i,gt}.$$

To perturb the translation vector $T_{i,gt}$ we add a random 3D vector ΔU to $T_{i,gt}$.

(24) $$T_{i}=T_{i,gt}+\Delta U.$$

To each set of RGB-D images obtained from a mesh model, five different rotational and translational perturbations are applied with rotation angles $2^{\circ }$ to $10^{\circ }$ and translation lengths 5 to 25 cm, respectively. Therefore, each set is perturbed in 10 different ways in our experiments.

## 6. Results

This section provides experimental results. We first evaluate our pairwise pose refinement algorithm on our synthetic dataset. The multi-view performance of the pose and depth refinement algorithms is then evaluated on the same dataset. Finally, the proposed method is applied to a real-world dataset acquired with active stereo cameras with similar parameters to our virtual ones.

We compare the proposed pose refinement algorithm to three existing algorithms using color information. We implemented the first two algorithms [8,10] performing a 6D search in the correspondence-search step. The algorithm of Johnson and Kang [8] (referred to as Color 6D ICP) is equivalent to our algorithm with K=1 and $M_{i,j}=I$. The algorithm of Korn et al. [10] (referred to as Color 6D GICP) uses a plane-to-plane cost function. The algorithm of Park et al. [24] (referred to as Color 3D ICP) performs the conventional 3D search and minimizes a cost function based on both color and depth differences across point clouds. We use the authors’ Open3D implementation [38].

The proposed algorithm is also compared to ICP algorithms that do not use color information. We use the Open3D implementations of the point-to-point ICP algorithm [5] (referred to as ICP (point to point)) and the point-to-plane ICP algorithm [6] (referred to as ICP (point to plane)). We implemented the algorithm of Segal et al. [13] (referred to as GICP) based on a plane-to-plane cost function.

We applied the adaptive thresholding scheme described in Section 3.1 to all the algorithms. We also applied the coarse-to-fine scheme [24] to all our implemented algorithms, i.e., Color 6D ICP, Color 6D GICP, and GICP. Thus, Color 6D ICP and our algorithm with K=1 differ only at the highest resolution. Likewise, the major difference between Color 6D GICP and our algorithm with K=1 is at the highest resolution. To evaluate the effectiveness of the coarse-to-fine scheme, we also implemented Color 6D ICP without the coarse-to-fine scheme. This method is referred to as Color 6D ICP (original).

The proposed algorithm is also compared to the Open3D implementation [38] of a state-of-the-art global registration algorithm [21] (referred to as FGR). The pose parameters obtained by the global registration algorithm are not affected by the initial alignment.

Finally, we can imagine an ideal method (referred to as Ground truth pose) that can estimate the ground truth pose. Using the ground truth pose in our synthetic dataset, we report such an ideal performance, which sets the theoretical goal of the real algorithms.

### 6.1. Pairwise Pose Estimation

In this experiment, we register a source point cloud to a reference point cloud by applying the pairwise registration algorithms. In this subsection Ours refers to the proposed pose refinement algorithm. The source view index *s* is increased from 1 to 11 and the reference view index *r* is set to s−1. The ground truth pairwise transformation is given by $R_{gt}=R_{r,gt}^{T}R_{s,gt}$ and $T_{gt}=R_{r,gt}^{T}\left(T_{s,gt}-T_{r,gt}\right)$. According to the type of perturbation, the initial pose parameter is set to either $R_{r,gt}^{T}\Delta QR_{s,gt}$ and $T_{gt}$ or $R_{gt}$ and $T_{gt}+R_{r,gt}^{T}\Delta U$, perturbing the source pose only.

The accuracy of registration is measured by computing the root mean square error (RMSE) between the registered source point cloud and the ground truth source point cloud:

(25) $$\mathrm{RMSE}=\sqrt{\frac{1}{N_{s}}\sum_{i=1}^{N_{s}}∥R_{gt}X_{i,gt}^{\left(s\right)}+T_{gt}-RX_{i}^{\left(s\right)}{-T∥}^{2}},$$

where $X_{i,gt}^{\left(s\right)}$ is the ground truth source point corresponding to $X_{i}^{\left(s\right)}$, with the same pixel location. If any of the two points is missing then the pair is discarded from the RMSE computation.

**Figure 4:**
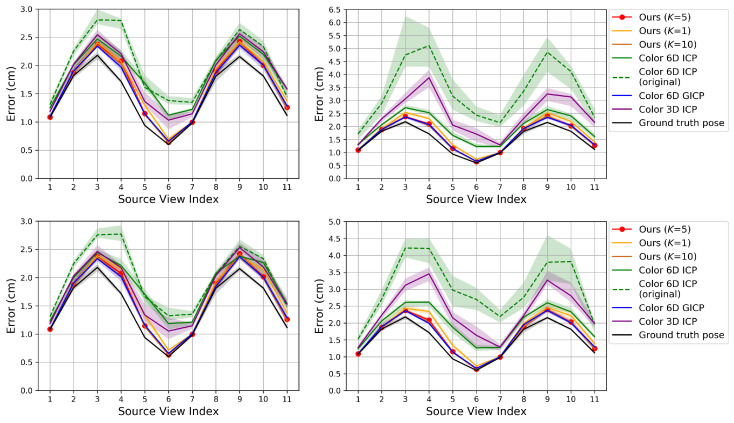
Evaluation of pairwise registration algorithms on our synthetic dataset. The proposed algorithm is compared to prior algorithms that use color. The algorithms are initialized with transformations that are perturbed away from the true pose. **Top**: Error according to the source view index with perturbation levels $2^{\circ }$ (**left**) and $10^{\circ }$ (**right**) in the rotational component. **Bottom**: Error with perturbation levels 5 cm (**left**) and 25 cm (**right**) in the translational component. The plot shows the median RMSE at convergence (bold curve) and the 40–60% range of RMSE across trials (shaded region). Lower is better. Best viewed in color.

**Figure 5:**
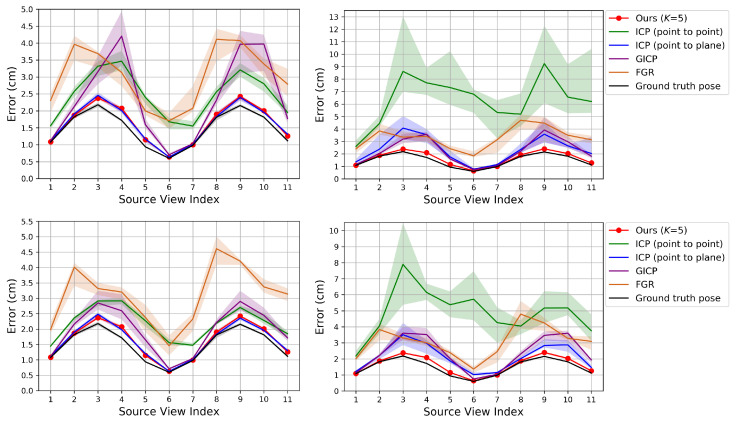
Evaluation of pairwise registration algorithms on our synthetic dataset. The proposed algorithm is compared to prior algorithms that do not use color. **Top**: Perturbation levels $2^{\circ }$ (**left**) and $10^{\circ }$ (**right**). **Bottom**: Perturbation levels 5 cm (**left**) and 25 cm (**right**).

**Figure 6:**
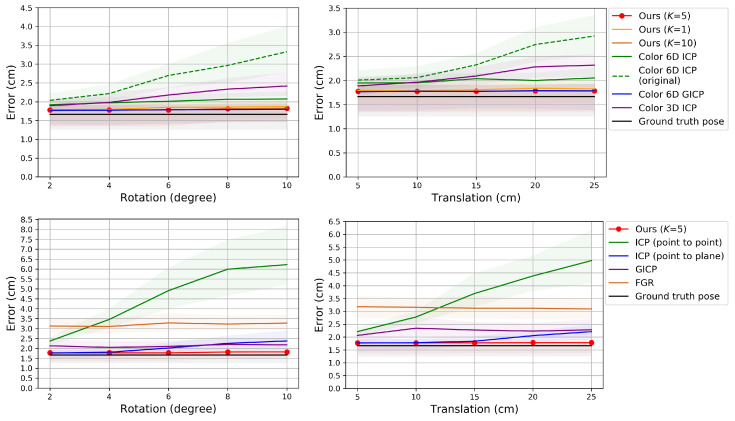
Evaluation of pairwise registration algorithms on our synthetic dataset. Error according to different perturbation levels in the rotational (**left**) and translational (**right**) components.

Figure 4, Figure 5 and Figure 6 show the RMSE. The proposed method and Color 6D GICP show the highest performance near the theoretical limit. In our method, setting *K* to 1 gives as competitive results as setting *K* to 5. However, the performance gap becomes larger as the perturbation level gets higher. On the other hand, setting *K* to a larger number, for example, 10, gives almost the same results as setting *K* to 5. It is also observed that the 6D search-based methods (Ours, Color 6D ICP, Color 6D GICP) show higher performances than the 3D search-based methods (ICP, Color 3D ICP). The comparison between cost functions is compatible with prior experiments [10,13]. The plane-to-plane cost function (Color 6D GICP) is the highest performer; however, the performance gap is reduced by increasing *K* of the point-to-plane cost function (Ours (K=5)). Finally, this experiment shows the effectiveness of the coarse-to-fine scheme [24], which has not been fully discovered yet. The methods using the coarse-to-fine scheme (Ours, Color 6D ICP, Color 6D GICP, Color 3D ICP) are robust to large perturbations, giving consistent errors for different perturbation levels. This is evidenced by the higher performance of Color 6D ICP than Color 6D ICP (original) irrespective of the perturbation level. The global registration method, FGR, is also robust to large perturbations; however, the RMSEs of the local registration algorithms based on the coarse-to-fine scheme are much lower than those of FGR.

In Figure 4 and Figure 5, most of the graphs look like sinusoids. This is due to the different distances from the object to the cameras, as shown in Figure 2. The farther cameras suffer from more depth errors than the closer cameras, and the registration errors are proportional to the depth errors in the ideal case. Thus, source views 3 and 9 have the highest RMSE while source view 6 has the lowest RMSE.

The running time of the pairwise registration algorithms is reported in Table 1. The running time was measured on a computer running Ubuntu 18.01 with an AMD Ryzen Threadripper 1920X 12-core processor and 128 GB of RAM. In Table 1, the first five algorithms use our unoptimized Python implementation of the Gauss–Newton algorithm while the core of the last four algorithms is written in C++. Therefore, a direct comparison is not fair across the two different implementations. The running times of GICP and Color 6D GICP are slightly shorter than Ours (K=1), although the computation of $M_{i,j}$ in GICP and Color 6D GICP requires more operations than in our algorithm. We conjecture that the faster convergence of the two algorithms reduces the running time. The running time of our algorithm is not linear with *K*. The running time of Ours (K=5) is about twice as long as Ours (K=1).

### 6.2. Multi-View Point Cloud Registration

In this experiment, we evaluate the multi-view performance of the algorithms. For comparison, we apply the existing pairwise registration algorithms to Algorithm 3 so that they can replace the role of our pose refinement algorithm. Algorithm 4 is then applied to the merged point cloud to reduce the registration error further.

The accuracy is measured by computing the RMSE between the registered source point cloud and the ground truth source point cloud:

(26) $$\mathrm{RMSE}=\sqrt{\frac{1}{N_{s}}\sum_{i=1}^{N_{s}}∥R_{s,gt}X_{i,gt}^{\left(s\right)}+T_{s,gt}-R_{s}X_{i}^{\left(s\right)}-T_{s}{∥}^{2}}.$$

Unlike Equation (25), $R_{s,gt}$ and $T_{s,gt}$ are directly used as the ground truth pose parameters.

**Figure 7:**
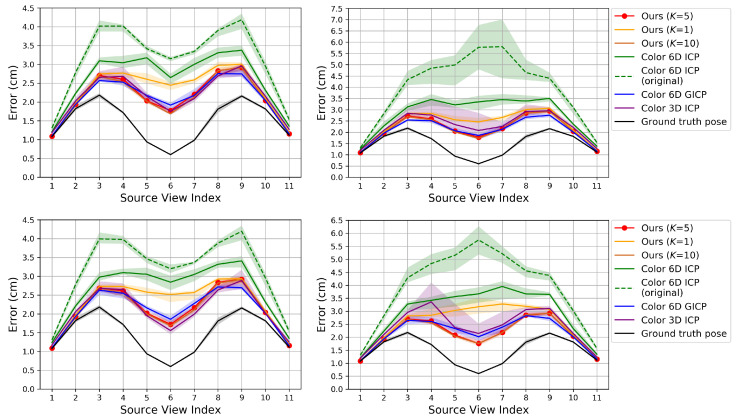
Evaluation of multi-view point cloud merging algorithms on our synthetic dataset. The proposed algorithm is compared to prior algorithms that use color. **Top**: Perturbation levels $2^{\circ }$ (**left**) and $10^{\circ }$ (**right**). **Bottom**: Perturbation levels 5 cm (**left**) and 25 cm (**right**).

**Figure 8:**
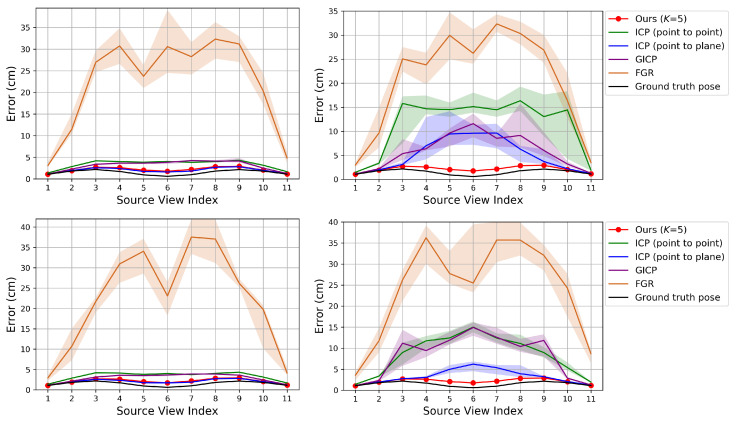
Evaluation of multi-view point cloud merging algorithms on our synthetic dataset. The proposed algorithm is compared to prior algorithms that do not use color. **Top**: Perturbation levels $2^{\circ }$ (**left**) and $10^{\circ }$ (**right**). **Bottom**: Perturbation levels 5 cm (**left**) and 25 cm (**right**).

**Figure 9:**
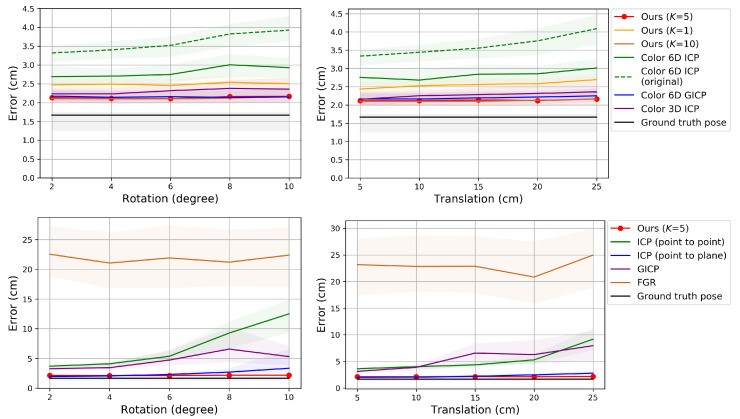
Evaluation of multi-view point cloud merging algorithms on our synthetic dataset. Error according to different perturbation levels in the rotational (**left**) and translational (**right**) components.

Figure 7, Figure 8 and Figure 9 show the RMSE. The proposed algorithm and Color 6D GICP show the highest performance. The performance of Color 6D GICP is slightly higher for the high rotational perturbation, while the performance of our algorithm is slightly higher for the high translational perturbation. In this experiment, the performance gap from the ideal method (Ground truth pose) becomes more prominent as the viewpoint gets farther from the reference viewpoint. This performance degradation is due to the accumulation of the pairwise registration error.

In our method, setting *K* to 1 is not so competitive as setting *K* to 5 anymore due to the error accumulation. Like the results in Section 6.1, setting *K* to 10 gives similar results to setting *K* to 5. The multi-view performance of Color 3D ICP is higher than Ours (K=1) although Ours (K=1) shows a higher pairwise performance. We conjecture that this is due to the advanced cost function of Color 3D ICP [24].

In Figure 8, the performance of ICP (point to plane) is slightly higher than our algorithm for the low perturbations. Since ICP (point to plane) does not use the coarse-to-fine scheme [24], we conjecture that our original fine-resolution algorithm (Algorithm 1) will also find a more accurate pose from such an accurate initial pose. However, in practice, it is not easy to predict the perturbation level. Therefore, we chose to use the coarse-to-fine scheme for the robustness.

FGR gives the worst results in Figure 8. Algorithm 3 gradually improves the accuracy of the poses using their previous estimates. Since FGR is not affected by the initial or previous alignments, Algorithm 3 can hardly improve the accuracy if FGR is used as its pairwise registration method. Even though FGR shows the state-of-the-art performance for global registration problems as in [24], our method based on the iterative *K*-closest point algorithm shows better performance for merging multi-view point clouds than other methods including FGR.

**Figure 10:**
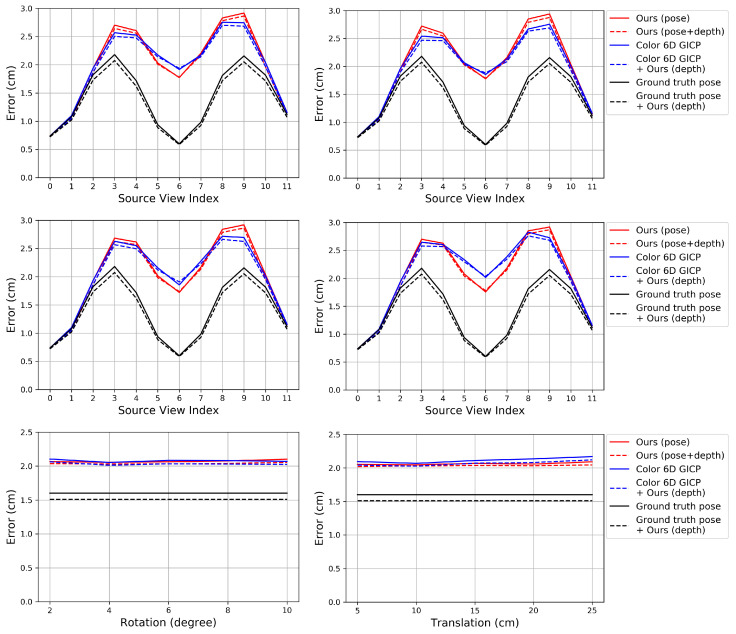
Evaluation of our depth refinement algorithm on our synthetic dataset. The proposed depth refinement algorithm is applied to the merged point cloud obtained by different methods. **Top**: Perturbation levels $2^{\circ }$ (**left**) and $10^{\circ }$ (**right**). **Middle**: Perturbation levels 5 cm (**left**) and 25 cm (**right**). **Bottom**: Error according to different perturbation levels.

Figure 10 shows the RMSE after applying the proposed depth refinement algorithm to the merged point clouds obtained by different methods. In Figure 10, Ours (depth) refers to the depth refinement algorithm. For the three methods, Ours (depth) reduces the registration error further. If the pose is more accurate (Ground truth pose) then the improvement is greater.

**Figure 11:**
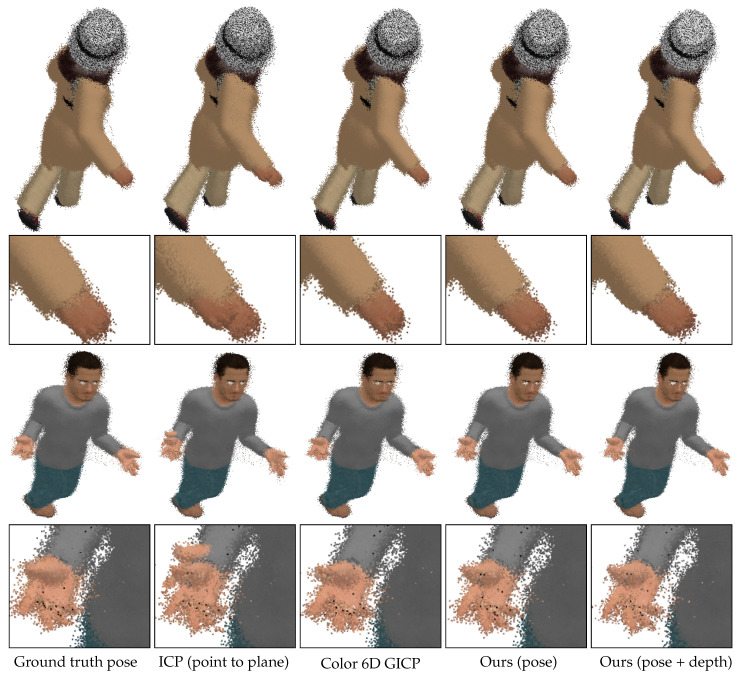
Point cloud rendering results. **First and third rows**: Merged point clouds. **Second and fourth rows**: Magnified hand regions. We note that neither a preprocessing nor a postprocessing method has been applied to the results.

Figure 11 shows merged point clouds obtained by different methods. The sample results have been obtained from the inputs with 25 cm perturbation levels. To show the difference between the methods, we did not apply any preprocessing or postprocessing methods. Color 6D GICP and our pose refinement algorithm (Ours (pose)) achieve visually similar alignments to that of Ground truth pose. It can be observed that the noisy points are moved toward the surfaces by our depth refinement algorithm (Ours (pose+depth)), achieving closer alignments. The proposed method improves the visual quality of the merged point cloud as well as reduces the registration error.

The average running time of Algorithm 4 is 596.80 s and the average running time per a pair of $S_{s}$ and $S-{\hat{S}}_{s}$ is 25.95 s. The running time was measured with the same computer used in the previous subsection.

### 6.3. Application to a Real-World Dataset

**Figure 12:**
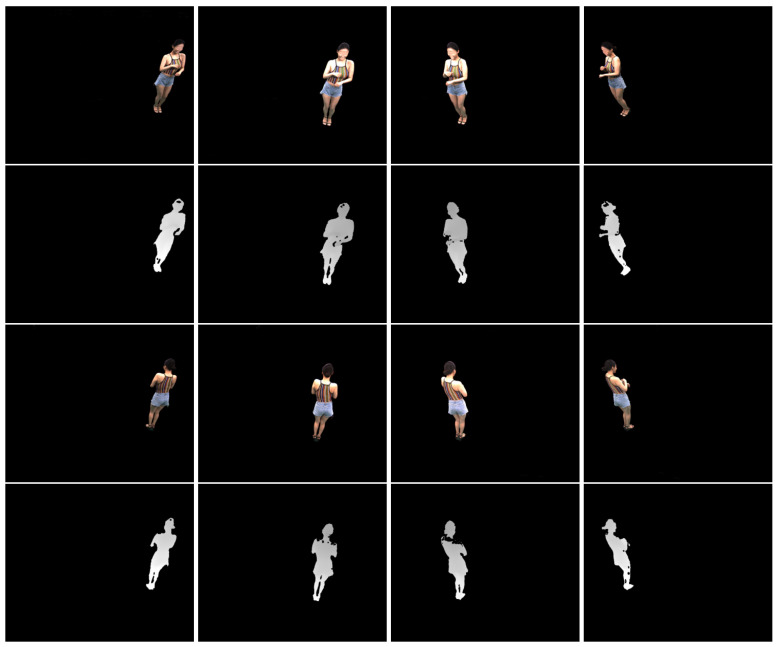
Real multi-view RGB-D images. **First and third rows**: Color images of the model. **Second and fourth rows**: Depth images of the model. The intensity of the depth images is linear with depth values. The face regions in the front views have been blurred to protect the model’s privacy.

The proposed algorithms are applied to a real-world dataset acquired with active stereo cameras with similar parameters to ours. The dataset is composed of eight RGB-D images as shown in Figure 12. The depth data was estimated by applying the PatchMatch stereo matching algorithm [39] followed by the left-right consistency check. As postprocessing, we applied a 3 × 3 median filter to the depth data and warped the 3D points to the color image coordinate frames. The 3D points projected to the background regions were then removed. Since the dataset has not been captured with accurate laser scanners, an exact quantitative evaluation is not available. The overlap between neighboring views is not regular but the calibration is more accurate than the worst cases of our experiments in the previous subsections. Thus, each source point cloud $S_{s}$ is registered to $S-{\hat{S}}_{s}$ in this application to establish a sufficient number of point correspondences.

**Figure 13:**
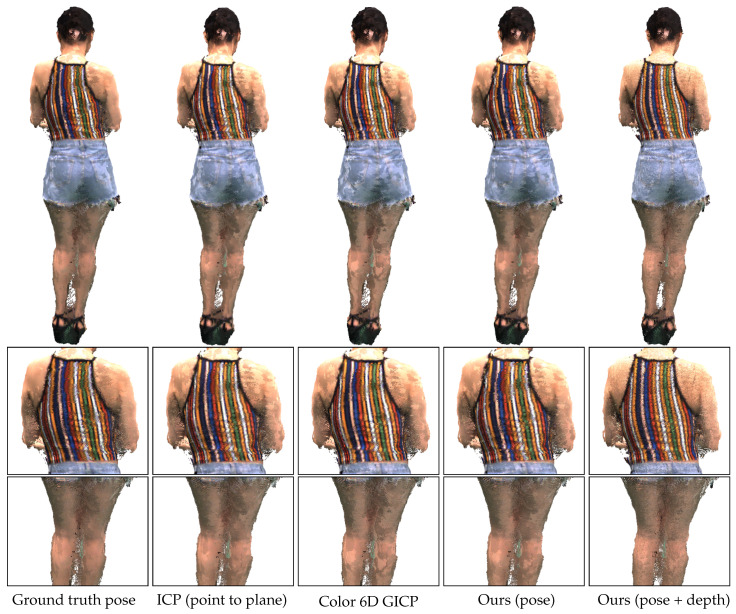
Point cloud rendering results. **First row**: Merged point clouds. **Second row**: Magnified regions in which our depth refinement algorithm produces improved results. **Third row**: Magnified regions in which our depth refinement algorithm produces artifacts. We note that no postprocessing method has been applied to the results.

Figure 13 shows merged point clouds obtained by different methods. Due to the accurate calibration, ICP (point to plane) gives visually more accurate results than Ours (pose). This result is compatible with the low-perturbation results in Figure 8.

Our depth refinement algorithm recovers from the worst alignment of Ours (pose). Ours (depth) corrects the stripes on the back to preserve their parallel structure. The large stain on the shoulders of the merged point clouds is due to the rendering of different surfaces that are not perfectly aligned. The stain size has been reduced in the result of Ours (pose+depth) due to the closer alignment.

On the other hand, Ours (depth) produces some flying points near the outer thighs as shown in the third row of Figure 13. There can be several reasons for those artifacts, including inaccurate estimation of the surface normals and numerical instability of the Gauss–Newton algorithm.

## 7. Conclusions and Future Work

We proposed iterative *K*-closest point algorithms that minimize a cost to align two colored point clouds. The cost function is based on color-supported probabilistic correspondences and point-to-plane distances. Our first algorithm regards the pose parameters of a point cloud as variables and finds the minimizer of the cost. In our second algorithm, we regard the measured depth values as variables and derive an update equation for refining the depth values. The experiments on our synthetic dataset demonstrated that our pose refinement algorithm outperforms prior registration algorithms, showing as high performance as our coarse-to-fine implementation of the state-of-the-art algorithm. Our depth refinement algorithm was shown to achieve more accurate and closer alignments from the outputs of the pose refinement algorithms.

Through the experiments, we found the following results. We found that using one-to-many correspondences (K=5) is more accurate than using one-to-one correspondences (K=1). We also found that the coarse-to-fine scheme along with the 6D search gives robust and accurate results.

To improve the numerical stability of our depth refinement algorithm, we can employ a more advanced cost function or a more advanced optimization algorithm. For example, the plane-to-plane cost function, which performed higher than the point-to-plane cost function, is a good candidate. In our future research, we are going to investigate different variants of our depth refinement algorithm to reduce the artifacts.

## Figures and Tables

**Table 1:** Average running time (s).

Ours (*K* = 10)	47.33
Ours (*K* = 5)	33.34
Ours (*K* = 1)	17.92
GICP	16.49
Color 6D GICP	15.20
Color 6D ICP	1.20
Color 6D ICP (original)	8.68
ICP (point to plane)	0.89
ICP (point to point)	0.86
Color 3D ICP	0.38
FGR	0.07

## Abbreviations

The following abbreviations are used in this manuscript:

ICP	Iterative Closest Point
RGB-D	Red Green Blue—Depth
IR	Infrared
GICP	Generalized ICP
RMSE	Root Mean Square Error
